# A Minireview for Recent Development of Nanomaterial-Based Detection of Antibiotics

**DOI:** 10.3390/bios13030327

**Published:** 2023-02-27

**Authors:** Jiafu Hong, Mengxing Su, Kunmeng Zhao, Yihui Zhou, Jingjing Wang, Shu-Feng Zhou, Xuexia Lin

**Affiliations:** 1 Department of Chemical Engineering & Pharmaceutical Engineering, College of Chemical Engineering, Huaqiao University, Xiamen 361021, China; 2 State Key Laboratory for Marine Corrosion and Protection, Luoyang Ship Material Research Institute, Xiamen 361101, China

**Keywords:** antibiotic, nanomaterials, electrochemical, optical technique

## Abstract

Antibiotics are considered a new type of organic pollutant. Antibiotic residues have become a global issue due to their harm to human health. As the use of antibiotics is increasing in human life, such as in medicine, crops, livestock, and even drinking water, the accurate analysis of antibiotics is very vital. In order to develop rapid and on-site approaches for the detection of antibiotics and the analysis of trace-level residual antibiotics, a high-sensitivity, simple, and portable solution is required. Meanwhile, the rapid nanotechnology development of a variety of nanomaterials has been achieved. In this review, nanomaterial-based techniques for antibiotic detection are discussed, and some reports that have employed combined nanomaterials with optical techniques or electrochemical techniques are highlighted.

## 1. Introduction

Antibiotics have been widely used for the prevention and treatment of bacterial infections in farming, animal husbandry, medical treatment and so on [1,2]. However, the extensive, ever-increasing use of antibiotics and instances in which antibiotics are not completely absorbed or completely metabolized create residues which flow into natural ecosystems, especially aquatic environments [3,4]. Various kinds of antibiotics have been detected in food and animals, polluting surfaces, ground water, and even drinking water [5]. Importantly, long-term exposure to residual antibiotics can increase resistant microorganisms and antibiotic resistance [6]. According to a report from the World Health Organization, antibiotic resistance causes approximately 700,000 people deaths every year and is considered a major threat to human health. Moreover, through food-chain transmission, the accumulation of antibiotics in environmental media and food products have side effects on human health, such as decreasing human immunity [7]. Furthermore, the residual antibiotics may also affect the sustainable development of society and the economy. The ever-increasing use of antibiotics and residual antibiotics causes antibiotic pollution, a problem which is becoming increasingly serious. Antibiotics are becoming a new type of organic pollutant in the environment. Therefore, it is of extreme importance that antibiotics are controlled for the protection of human health and safety.

There are currently technologies that have been developed for the qualitative/quantitative analysis of antibiotics [8,9]. These technologies mainly include two categories. The first is the precision instrument analysis method, represented by chromatography–mass spectrometry, which requires special instruments and equipment and professional personnel to operate has a high detection cost [10]. The other category comprise rapid detection methods, mainly including the enzyme-linked immunosorbent assay (ELISA), electrochemical methods, optical methods, and biosensor methods [11,12]. Rapid detection methods have become hotspots in recent years because of their remarkable advantages such as portability, fast speed, low cost, etc. However, for rapid and highly accurate antibiotic analytical methods, some huge challenges are still encountered. One difficulty are high backgrounds and multi-components due to complex matrices, limiting accuracy and sensitivity [13]. Second, antibiotics are easy to migrate and transform in their surrounding environment, so it is a challenge to maintain their state and form [14]. Third, sensitivity is a major challenge for antibiotic detection owing to their low concentration in aquatic environment samples or food samples [9]. Therefore, there are great demands to develop simple, fast, sensitive, and cost-effective detection technologies for antibiotics.

A biosensor is a powerful tool for detecting and measuring antibiotics in medical diagnostics. It involves the dynamics and thermodynamics of antibiotic–antibody interaction or the detection of antibiotics. As proper transducer converts, antibiotic biosensors are usually designed by immobilizing a biological element, such as deoxyribonucleic acid (DNA), ribonucleic acid (RNA), or antibodies on the surface of nanomaterials. These biosensors can covert the bio-chemical signal into measurable signals. Due to the development of technology, most antibiotic biosensor research mainly focuses on label-free, on-site and real-time detection. Every analysis required antibiotic concentration, required accuracy, analytical time, reuse times, and cleanliness. Coupled with nanomaterials, antibiotic biosensors are developed for a faster, more selective detection with automation and ease of operation.

**Figure 1:**
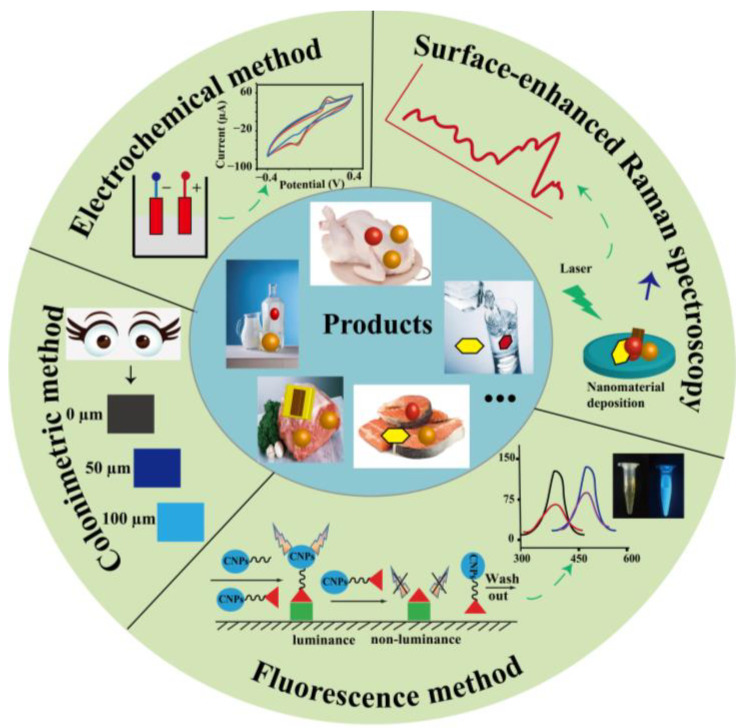
Nanomaterial-based methods for the determination of antibiotics.

Nanomaterials can enhance the interaction between biosensors and antibiotics. As a result of advances in nanotechnology development, different kinds of nanomaterials, including metal nanomaterials, silica nanoparticles, and carbon nanomaterials, have been developed [15,16,17]. Due to their electrochemical activity, photostability, and versatile surface modification, scientists have explored various kinds of methods for the trace detection of antibiotic residues based on nanomaterials as electrochemical events or luminous probes in Figure 1 [18,19]. Most applications of nanomaterials for antibiotic analysis are low toxicity or non-toxic, and their surfaces are rich with multiple functions [20,21,22]. Multiple functions on the nanomaterial surface are often modified for use as electrodes or catalysts. In order to improve their selectivity and accuracy, nanomaterials are often coupled with an antibody or functional oligonucleotide. Nanomaterials have been recently well-developed to detect various antibiotics such as tetracycline, streptomycin, penicillin, and ampicillin in foods such as milk, drinking water, and other aquatic environments, aiding in environmental monitoring and food safety. Herein, we attempt to provide a mini-review of recent nanomaterials based on electrochemistry and optical technologies for the determination of antibiotics. In particular, this review surveys carbon nanomaterials or graphene and their derivatives to establish electrochemical and optical methods for the analysis of antibiotics.

## 2. Biosensors

Antibiotic biosensors often contained two parts: a receptor and a transducer. The receptors of antibiotics are often antibodies or aptamers. They can efficiently recognize and specifically capture the target antibiotic. A transducer, which could be developed using nanomaterials, can convert antibiotic–receptor interactions into measurable signals such as optical, electrochemical, and mass changes. In recent decades, many biosensors have been developed for the detection of antibiotic residues. For example, some researchers applied enzymes as receptors for the recognition of antibiotic targets. The most common enzymes are horseradish peroxidase [HRP], pencillinase [PCN], and glucose oxidase [GOx]. In order to analyze tetracyclines and streptogramins in human plasma, Kling et al. applied GO as an enzyme to label a repressor protein (TetR or PIP) that binds to a specific operator DNA [23]. In the presence of class-specific antibiotics, the proteins have conformational changes and cannot bind to the specific operator DNA. These proteins, which compete with tetracycline and streptogramin antibiotics, are a competitive assay for the quantification of tetracycline and streptogramin from clinically relevant samples. Based on this mechanism, researchers designed an electrochemical, microfluidic chip for tetracycline and streptogramin analysis. Based on specific antigen–antibody recognition of targets, other researchers have developed electrochemical immunosensors for antibiotic residue analysis. Zhao et al. immobilized a kanamycin antibody onto a composite film for the detection of kanamycin. In order to decrease the limit of detection, Prussian blue was selected as an electron transfer mediator and was modified onto the electrode with a water-soluble graphene sheet through electrostatic adsorption. Nanoporous gold was then immobilized onto the as-prepared electrode to capture the kanamycin [24]. In addition, aptamer-based biosensors have emerged as a robust detection strategy for antibiotic residues. Compared to antibodies, aptamers have received tremendous attention in antibiotic residue analysis because they have physicochemical stability, are low-cost, easy to synthesize in-vitro, and have simple modification procedures. Lin’s group exploited the aptasensors to achieve a quantitative ampicillin and kanamycin assay [25,26]. The aptasensors were designed using their aptamers and a secondary DNA fragment. Moreover, a non-specific DNA was used as an internal standard to decrease non-specific adsorption. Finally, molecular imprinted technologies have gained increasing attention as antibiotic sensors developed for the presence of specific receptor sites for target antibiotic molecules. The mechanism operates through a sacrificial spacer method that involves the polymerization of cross-linkers and functional monomers with antibiotic molecules. Regardless, transducer mediators in every biosensor should be incorporated into the selectivity analysis. Moreover, the sensitivity of the developed biosensors also needs to be improved for the determination of antibiotic residues. Therefore, significant efforts have been made toward developing nanomaterials for highly sensitive and selective antibiotic detection.

## 3. Nanomaterial-Based Electrochemical Analysis of Antibiotic 

### 3.1. Electrochemical Analysis for Antibiotics

As antibiotic abuse became increasingly familiar, concerns about the harm from antibiotics to humans and the environment grew. People also became concerned about the effect of antibiotics on the ecosystem [1,3,6]. Therefore, the detection of antibiotic residues is a matter of urgency. It was discovered that electrochemical analysis is of great interest to researchers due to its simplicity of operation, rapid analysis, low cost of instrumentation, the possibility of in-situ analysis, and high sensitivity. The core of electrochemical analysis is the placement of biosensors on a working electrode. Although antibodies or oligonucleotides in analytical sensors can specifically recognize and capture target antibiotic molecules, one of electrochemical antibiotic detection mechanism is based on the electro-oxidation of antibiotics [27,28,29]. Therefore, the selectivity and sensitivity of electrochemical analysis are highly dependent on the activity of the biosensor on the electrode surface. To date, antibiotic electrochemical methods include direct electrochemical analysis and indirect electrochemical analysis. The direct electrochemical analysis method is often based on the antibiotics themselves and their redox groups, and can generate the redox signal on the electrode using the redox groups. A linear relationship can be found between the strength of the electrochemical signal and the concentration of the antibiotics. Indirect electrochemical analysis is often established due to antibiotics lacking a redox signal; however, the antibiotics can be captured by antibodies or oligonucleotides or reacted with metal ions or other substances to generate electrochemical signals on modified electrodes. Since the electrical signal is proportional to the concentration of the antibiotic in a certain range, a quantitative analysis can be performed. In addition, the use of modified electrodes is a common method to determine the concentration of antibiotics without direct electrochemical signals.

### 3.2. Nanomaterial-Based Electrochemical Method of Antibiotic

**Figure 2:**
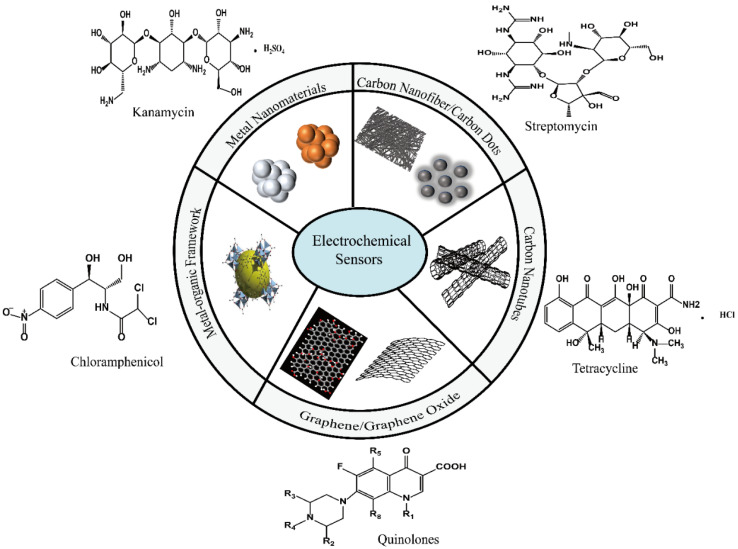
Nanomaterials for electrochemical method of antibiotic.

Among antibiotic electrochemical methods, the voltammetry method is usually used for qualitative analysis, while anodic stripping voltammetry (ASV) or the linear sweep voltammetry (LSV) technique are often used to quantitative analysis. For antibiotic electrochemical analysis, electrochemical redox signals and the rate of the oxidation reaction are mainly dependent on the electrocatalytic activity and the electron transfer kinetics of the biosensors. The developed biosensors often contained antibodies or aptamers with nanomaterials. As part of biosensors, nanomaterials were widely used as electrode materials for their tunable size, large specific surface area, high catalytic activity, and special electrical properties for antibiotic analysis in Figure 2. Additionally, biosensors can enhance the interaction with detection probes and work electrodes, which can be used not only for signal amplification but also to capture targets. Several carbon-based materials containing graphite, carbon nanofibers, carbon nanotubes, fullerenes, graphene, and others have been applied to established electrochemical sensors [23,24]. Some metal nanomaterials, such as gold nanoparticles (AuNPs), silver nanoparticles (AgNPs), and metal–organic frameworks (MOFs), are also commonly used as electrode materials [8,30].

Our current society requires faster detection and more sensitivity. A novel nanomaterial-based electrochemical sensor often can be developed with signal transduction events. The required faster electrochemical sensor needs a faster electron transfer rate. In order to improve the electron transfer rate, various kinds of nanomaterials were developed and modified. For example, scientists have changed the proportion of cerium and oxygen to obtain different electron transfer rates of cerium oxide nanomaterials, resulting in and changing the composition of nanomaterials [31]. Morphology, dimensions, crystal structure quality, and crystallographic axis orientation are also altered nanomaterial characteristics [32]. Due to the development of nanomaterials, the researchers are trying to improve electrochemical analysis of antibiotic detection with higher sensitivity and selectivity, or faster speed. In addition, pollution has long plagued researchers for the development of electrochemical methods, especially for electrode contamination. Through the application of nanomaterials, self-cleaning nanomaterials have also been gradually developed and used for the modification of work electrodes. Zhu et al. developed a self-cleaning electrode based on the application of superhydrophobic and conductive nanocomposite for the construction of a working electrode [29]. Owing to the superhydrophobicity of polydimethylsiloxane (PDMS), the electrode avoids both the adsorption of molecules and oxidation in air. Through the application of high-conductivity materials such as multi-walled carbon nanotubes (MWCNTs), electrochemical signals are enhanced. Furthermore, Zhang et al. also developed self-cleaning electrodes for the simultaneous detection of adrenaline, serotonin, and tryptophan [33]. On the basis of comprehensive studies on nanomaterials, researchers have applied nanomaterials not only for saving analytical time and improving sensitivity but also for the fabrication of self-cleaning and refreshable electrochemical sensors.

#### 3.2.1. CNT-Based Electrochemical Method of Antibiotic 

**Figure 3:**
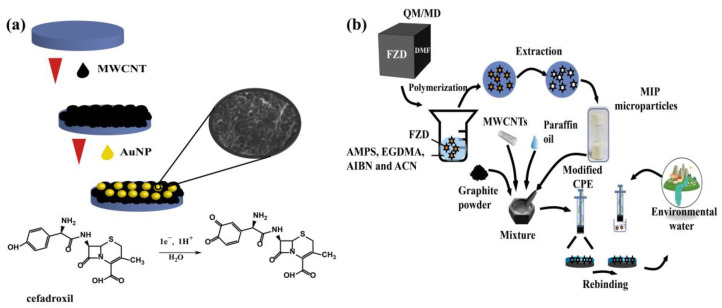
(**a**) The determination of cefadroxil β-lactam antibiotic was carried out using a modified gold nanoparticle/multiwalled glassy carbon nanotube [34]. (**b**) Molecularly imprinted carbon paste electrode for furazolidone detection [35].

Carbon nanotubes (CNTs) are one-dimensional nanomaterials which can be divided into single-walled carbon nanotubes (SWCNTs) and MWCNTs. Since the first synthesis from graphene (GR) sheets curled at a specific angle in 1991, CNTs have been widely used in environmental monitoring, medical diagnosis, marine protection, and biology. Due to their good biocompatibility, large specific surface area, high mechanical strength, and the unique advantages of stronger electrical properties and faster electron transfer ability, CNTs have attracted extensive attention with respect to developing antibiotic sensors. For the exploitation of CNTs in electrochemical sensors, their excellent electrical conductivity and electrochemical catalytic activity can be used. However, with a combination of CNTs and other nanomaterials, the sensor would demonstrate a better performance than a sensor based only on CNTs. Sanz’s group dispersed functionalized MWCNT in chitosan and decorated it with AuNPs. The prepared nanomaterials were then modified with glassy carbon electrodes. Finally, the developed electrodes were used to detect cephalexin in Figure 3a [34]. The limit of detection was low: 0.22 M. The modified electrode, which was based on MWCNTs and AuNPs, showed a 7-fold higher sensitivity than that of the bare electrode. These results demonstrated that the synergistic qualities of CNTs could improve the performance of electroanalytical sensors. The excellent ductility of CNTs provides more opportunities and possibilities for the development of portable electrochemical detection sensors. Due to their portable and simplicity, CNT-based antibiotic electrochemical sensors can be applied not only in food but also in the environment, such as sea water, aquaculture water, soil, and the atmosphere. Rebelo’s group constructed a selective, molecularly imprinted polymer (MIP) sensor for furazolidone (FZD) detection [35]. In this work, the FZD MIP microparticles were firstly prepared by precipitation polymerization. The work electrode was then constructed by the modification of the traditional carbon paste electrode (CPE) with MIP microparticles and multi-walled carbon nanotubes (MWCNTs), shown in in Figure 3b. The sensor showed a good linear response in the concentration range of 0.01 μM~1 μM. The detection limit was 0.03 μM. Moreover, the sensor was selective for furazolidone molecules with a 90% recovery. Additionally, the selectivity was also improved for the recognition of antibiotic molecules by the formation of an imprinted cavity.

#### 3.2.2. Graphene and Its Derivative-Based Electrochemical Analysis of Antibiotic 

**Figure 4:**
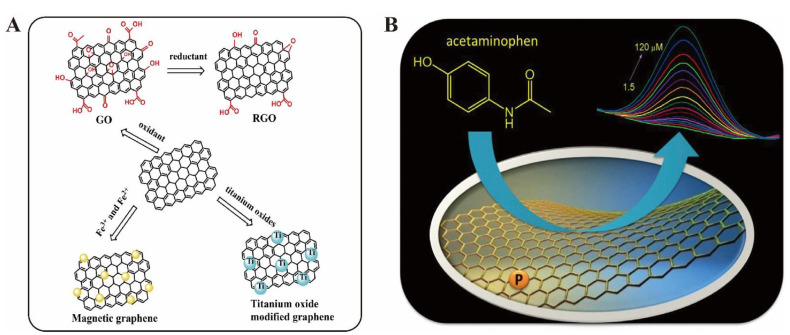
Electrochemical analysis for antibiotic detection based on graphene and its derivatives. (**A**) Functionalization of graphene [36]. (**B**) Phosphorus-doped graphene-based electrochemical sensor for sensitive detection of acetaminophen [37].

Carbon nanomaterial plays an important role in the fabrication of electrochemical devices for antibiotic detection. Among carbon nanomaterials and as 2D carbon nanomaterials with unique electronic and catalytic properties, GR and its derivatives have been widely used. GR has been fabricated by various methods including scotch-tape isolation, chemical vapor deposition, and chemical and electrochemical reduction. In order to enhance catalytic activity and electric conductivity, many atoms, such as O, N, S, and P, can be doped into GR. Furthermore, GR and its derivatives have a planar structure and can be easy modified, making them a promising functional material for the rapid analysis of antibiotics. Additionally, they have not only large specific surface area, excellent conductivity, and thermal conductivity but also an excellent electron transfer ability. These advantages have attracted extensive attention for use in developing antibiotic sensors. First, a single layer of GR and its derivatives can easily contact antibiotics through π-π interactions in Figure 4A [36]. Second, the porous structure and high surface area of GR and its derivatives can cause the faster diffusion or surface reaction of antibiotics, leading to a rapid and efficient analysis. Finally, the cost of GR and derivatives is low. In addition, they can be combined with organic and inorganic materials. The GR doping materials would provide antibiotic sensors with a faster electron transfer ability and better electrocatalytic activity, improving the sensitivity.

Graphene oxide (GO), which plays an important role in GR-based derivatives, is modified with various oxygen-containing groups. Although oxygen-containing functional groups of GO can reduce the electrical conductivity and limit its direct application as an electroactive material, it can be used by chemical modification or functionalization. In the past years, GO-based nanomaterials have obtained extensive attention in the development of electrochemical sensors. Xuan Zhang and his co-workers hydrothermally synthesized a phosphorus-doped graphene (P-RGO) material from GO and used the P-RGO to coat a glass carbon electrode (P-RGO/GCE). The prepared P-RGO/GCE electrode was then developed as a new electrochemical sensor for AP detection [37]. The application of P-RGO demonstrated that it can enhance electrochemical conductivity and accelerate electron transfer. Therefore, the modification of a glass carbon electrode with P-RGO showed an excellent electrocatalytic activity for the oxidation of AP. Under optimal conditions, a linear relationship for the AP was obtained in the range of 1.5~120 μM with a detection limit of 0.36 μM (S/N = 3). Although several types of sensors have been developed with a high selectivity, sensitivity is still a challenge in antibiotic electrochemical analysis.

To enhance the analytical performance of GR-family-based electrochemical sensors for the determination of antibiotics, GR family nanomaterials were combined with metal nanomaterials, metal oxide nanomaterials, and so on. Nehru et al. demonstrated the preparation of cobalt-doped Fe_3_O_4_ nanospheres deposited on graphene oxide (Co−Fe_3_O_4_ NS/GO), which were then used for voltammetry sensing for the detection of chloramphenicol in food [38]. The sensor had a detection limit of 1.04 nM and a sensitivity of 5.07 μA·μM^−1^·cm^−2^, and demonstrated a continuous, fast electrode performance, selectivity for chloramphenicol detection, cyclic stability, and reproducibility. For another example, Liu et al. prepared Ag-NPs functionalized with reduced graphene oxide (rGO) nanocomposites to establish electrochemical aptamer sensors on glass carbon electrodes for the sensitive and selective determination of the antibiotic chloramphenicol, shown in Figure 4B [39]. Under optimal conditions, the calibration curve was linear in the concentration range of 0.01 to 35 μM, and the detection limit was 2 nM. The excellent electrochemical characteristics of GO nanocomposites enable high sensitivity and selectivity for real-world sample analyses of antibiotics.

#### 3.2.3. Other Carbon Nanomaterial-Based Electrochemical Analysis of Antibiotic 

**Figure 5:**
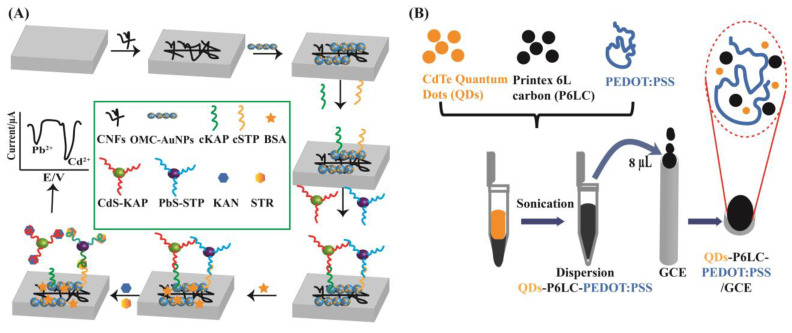
Nanomaterial-based electrochemical methods for antibiotic analysis. (**A**) The ultrasensitive electrochemical detection of kanamycin and streptomycin carbon nanofibers and mesoporous carbon-gold nanoparticles [40]. (**B**) An electrochemical device for the determination of amoxicillin based on a combination of nanomaterials such as Printex 6 L Carbon and cadmium telluride quantum dots within a poly(3,4-ethylenedioxythiophene) polystyrene sulfonate film [41].

In addition to CNTs and GR, other carbon nanomaterials such as carbon nanofibers (CNFs), carbon dots (CDs), and others have also attracted considerable interest in the development of electrochemical antibiotic sensors. In general, CNFs are cylindrical nanostructures which have a larger diameter and a longer length than CNTs. The application of CNFs for the construction of electrochemical sensors would improve the electron transfer performance because CNFs are stacked in sheet form with edge-plane defects. They can be activated on the entire surface. CNFs can also improve sensitivity by the expansion of the specific surface area of the electrode. In the exploitation of CNFs, mesoporous carbon, and AuNPs, Falan Li and his coworkers fabricated a screen-printed electrochemical electrode with an aptasensor for the ultrasensitive determination of kanamycin and streptomycin residues, shown in Figure 5A [40]. Coupled with AuNPs, mesoporous carbon, and CNFs, the aptasensor can be well-immobilized on the surface of the electrode. Thus, the fabricated electrochemical sensors had a high electrochemical conductivity and a high specific surface area and demonstrated a high stability and a selectivity to kanamycin and streptomycin with limits of detection (LODs) as low as 87.3 and 45.0 pM. Another work involved the use of printex 6 L carbon, cadmium telluride quantum dots, and poly(3,4-ethylenedioxythiophene) polystyrene sulfonate for the establishment of electrochemical amoxicillin sensors Figure 5B [41]. The synergy application of cadmium telluride quantum dots enhanced the electrochemical activity and the electron transfer rate and improved the electrode surface area, leading to enhancement of the anodic peak current for amoxicillin analysis.

Other carbon nanomaterials are carbon nanodots (CDs) and graphene quantum dots (GQDs). As a new class of nanomaterials, they have been widely employed. Pratik Kolhe fabricated an electrode through the application of GQDs for the determination of cephalexin based on the combination of cephalexin and bovine serum antibodies [42]. The fabricated electrode was optimized by Electrochemical Impedance Spectroscopy (EIS). With the developed electrode, cephalexin can be detected on-site and the limit of detection was low to 0.53 fM. However, although electrochemical methods are rapidly developing, some challenges still exist which require solutions based on nanomaterials [43]. Therefore, the synthesis of nanomaterials that are required more uniform, tunable in size, and a higher special surface area and are required to design novel functional groups on the surfaces of work electrodes. These will help us to further understand the relationships and mechanisms between nanomaterials and antibiotics. Methods of reducing cross interference, especially among structurally similar antibiotics, also need further study. Additionally, it is urgent to establish rapid, highly reliable on-site methods for antibiotics in a complex matrix. In addition, desirable, portable, automated devices may be developed, which might have great potential for further social demands.

## 4. Nanomaterial-Based Optical Analysis of Antibiotic 

Antibiotics have variety of types and interact with their background. In a complex environment, the structures of antibiotics can be changed, and the levels of antibiotics are often low. Therefore, rapid and sensitive methods of detecting antibiotics are required. In view of their excellent electronic, optical, and catalytic properties, nanomaterials have been powerful tools for developing technologies for the detection of antibiotics which enable highly sensitive, selective, and rapid analyses [18,19]. Optical sensors can interact with biorecognizable elements of targets and then transform them into optical signals. In recent years, nanomaterial-based antibiotic detection has been developed in combination with adsorption, Raman scattering, fluorescence spectroscopy, and luminescence spectroscopy [20,21,22]. Further development is in low-cost, rapid, portable, and environmentally friendly technologies for in-situ measurements. The integration of nanomaterial-based optical sensors could have great potential in the achievement of a very low detection limit. In addition, micro-sized sensors with nanomaterial-based sensors can be developed which will have benefits such as portability for in-situ measurements.

### 4.1. Nanomaterial-Based Fluorescence Analysis of Antibiotic 

**Figure 6:**
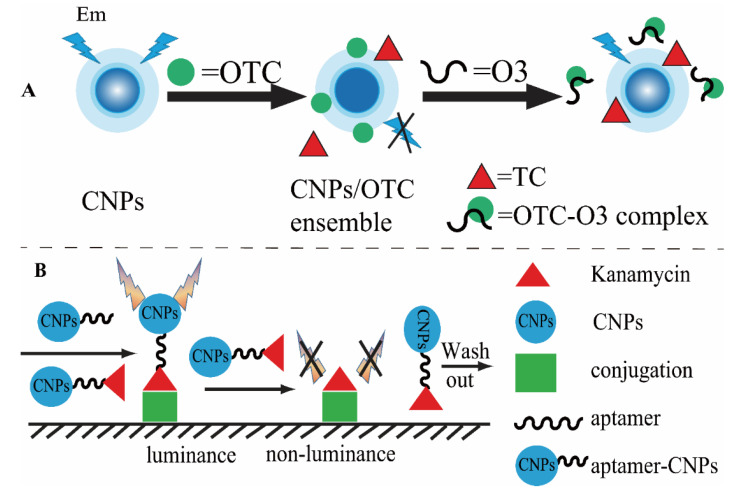
Carbon nanoparticle-based fluorescence methods for the determination of antibiotics. (**A**) Competitive analysis for oxytetracycline, based on aptamer and carbon nanoparticles [48]. (**B**) Microplate-based fluorescence method for kanamycin detection based carbon nanoparticles [49].

The rapid and sensitive assay of antibiotics is of great significance in public health, food safety, and environmental monitoring [5,44,45]. However, the sensitivity of antibiotic detection is a major challenge. In addition, to reduce analysis contamination, the amount of the sample should be as low as possible. Thus, it is highly desirable to develop methods that are not only highly sensitive but also simple, fast, practical, and involve low-cost protocols in routine detection. It is well-known that nanomaterials-based fluorescence detection methods exhibit a high sensitivity [46]. Among the large family of nanomaterial-based optical sensors, carbon nanoparticles are some of the most used [47]. Our group has established a competitive analysis for antibiotic residues based on carbon nanoparticles (CNPs) and oligonucleotide probes [48]. In this work, the CNPs can be quenched by oxytetracycline (OTC) and can recover the luminescence through the application of OTC oligonucleotide probes, shown in Figure 6A. This simple strategy was successfully applied to analyze OTC from drinking water with low relative standard deviations (RSDs), and the limit of detection was brought down to 0.002 ng/mL. Additionally, microplate-based fluorescence detection was also developed without the need for sophisticated laboratory equipment. As a particular advantage, the microplate assays have attracted increasing attention in residual antibiotic detection. Our group developed an ultrasensitive and highly selective CNP for the rapid detection of kanamycin residue through an aptamer-linked immunosorbent assay, shown in Figure 6B [49]. The established method can produce strong signals in a kanamycin assay for which the limit of detection was lower than 5.0 × 10^−8^ ppb. More importantly, the method was successfully applied to analyze kanamycin in milk with a simple sample treatment that has great potential for automation. Furthermore, the lateral flow immunoassay (LFA) was developed to meet demands due to the addition of a sample-separation procedure during the detection process. Honggui Lin and his co-workers developed a simple and rapid method based on LFA for the detection of antibiotics by labeling fluorescent CDs and aptamers [25,26].

### 4.2. Nanomaterial-Based Colorimetric Analysis of Antibiotic 

**Figure 7:**
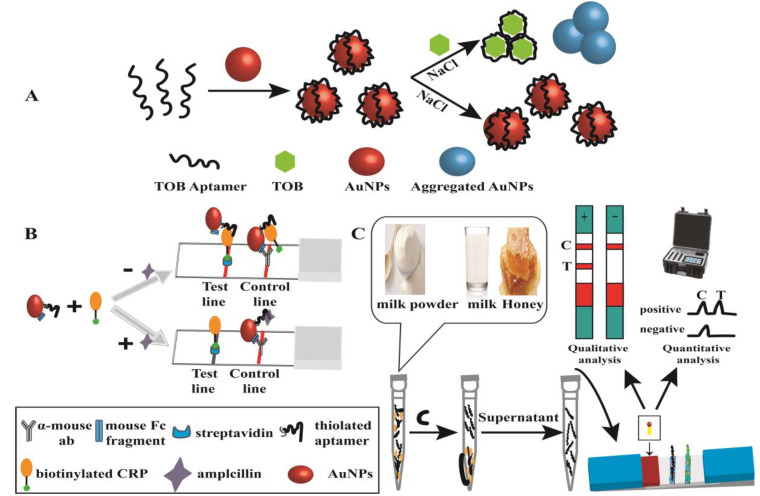
Nanomaterial-based colorimetric methods for antibiotic analysis. (**A**) The determination of tobramycin based on unmodified AuNPs, a single-strand DNA (ssDNA) probe, and AuNPs [53]. (**B**) Visual detection of ampicillin based on the combined of aptamer-based LFA and AuNPs [54]. (**C**) A fast and on-site detection of kanamycin, based on LFA with kanamycin-aptamer-modified AuNPs [55].

Colorimetric methods of antibiotic analysis have been developed due to their simple operation, fast detection, low cost, and portability [50,51]. Colloidal, spherical gold nanoparticles (AuNPs) are very popular for use in the colorimetric method [52]. The color change of a AuNP solute is based on the aggregation and dispersion of AuNPs. Therefore, AuNP-based methods often require detection probes to promote the aggregation and dispersion of AuNPs. Yuhong Xiang and his co-workers developed a method for the determination of tobramycin based on unmodified AuNPs, a single-strand DNA (ssDNA) probe, and AuNPs, shown in Figure 7A [53]. In the presence of TOB and the introduction of NaCl, a TOB aptamer can detach from the surface of the AuNPs and bind with TOB, promoting the aggregation of AuNPs and changing the solution color from red to purple-blue. However, in the absence of TOB and in the introduction of NaCl, the TOB aptamer cannot be detached from the surface of the AuNPs and the solution color remains red. This proposal was successfully used to detect TOB in milk and chicken eggs. Numerous AuNP-based antibiotic assays have been reported. However, the analysis accuracy needs to be improved. Therefore, an alternative strategy was reported by Lars Kaiser et al. for the detection of ampicillin based on the combination of an aptamer-based LFA and visual detection on-site with AuNPs, shown in Figure 7B [54]. Nandi Zhou’s group developed a fast and on-site detection of kanamycin based on an LFA with kanamycin-aptamer-modified AuNPs, shown in Figure 7C [55]. The sensitivity was improved by the application of oligonucleotide-DNA-modified silver nanoparticles. With advantages of high separation efficiency, simple operation, and visualization, many LFA method for antibiotic analysis rely on antibodies or aptamers to generate color changes with AuNPs. These proposals have great potential applications in healthcare, medicine, immunology, food safety, environmental evaluation, and others.

### 4.3. Nanomaterial-Based Surface-Enhanced Raman Spectroscopy for Antibiotic Analysis

The identification and quantity analysis of antibiotics from food and liquid samples are complex and time-consuming due to a great variety of antibiotics and complex matrices. Many efforts have been devoted to this cause. Surface-enhanced Raman spectroscopy (SERS) is the enhancement of the Raman signals of molecules adsorbed on the surface of a metal substrate. It can provide unique fingerprint spectrograms of targets and can simultaneously detect multiplex antibiotics. SERS is a powerful technology for the determination of antibiotics [56]. Hongji Li et al. developed an Ag nanoparticle (AgNP)-based SERS membrane for the high-sensitivity detection of antibiotics [57]. The hydrophilicity of the SERS membrane was greatly improved by the addition of the AgNPs. Under optimal conditions, the developed SERS membrane can detect antibiotics in the range from 1.0 nmol/L to 200 nmol/L. Recently, increasing attention has been paid to coupling AuNP-based biosensors with SERS for rapidity, simplicity, and economy. AuNPs can be self-assembled at the oil–water interface and can be used in the application of SERS biosensors. Weirong Yao’s group developed AuNP-based SERS for the rapid detection and characterization of furadantin and furaltadone in food samples [58]. The developed SERS can be qualitative analyzed for furadantin and furaltadone by their SERS peaks at 1420 and 1456 cm^−1^. The quantitative analyses for furadantin and furaltadone were undertaken at 1008 and 1162 cm^−1^. Under optimal conditions, the limit of detection could be as low as 5 ppm. Wang group have developed a rapid and sensitive SERS method for monitoring Penicillin G residue in milk. The developed method exploited a silver shell, surrounding the core gold nanoparticles with inositol hexaphosphate [59]. Additionally, a sensitive SERS coupled with lateral flow immunochromatography assay could also be developed using metal nano-stars.

## 5. Conclusions

Antibiotic residues have an important effect on human health and the ecological environment. To reduce the abuse of antibiotics and to control residual antibiotics, the accurate detection of antibiotics or residual antibiotics is greatly demanded. However, due to the complexity of samples such as milk, apples, and wastewater, the detection of residual antibiotics at a trace level remains difficult. Nanomaterial-based techniques are promising for antibiotic detection because of their unique advantages, including their low cost, biocompatibility, stable properties, good catalytic performance, high specific surface area, and self-cleaning ability. Meanwhile, nanomaterials have been successfully utilized in developing ultrasensitive antibiotic detection methods. As highlighted in this review, nanomaterials have been successfully integrated with a variety of detection techniques, such as electrochemical technology, fluorescence technology, surface-enhanced Raman methods, and colorimetric methods. To develop portable devices, LFA and electrochemical technologies have been integrated with biosensors, offering a promising strategy to separate and detect trace-level antibiotics in complex samples. We anticipate that the developing techniques with nanomaterials will become a powerful tool in environmental monitoring, food safety, and medical diagnosis, as well as other fields. Furthermore, we believe that nanomaterial-based antibiotic analysis methods with a higher sensitivity, higher speed and integration, increased portability, and increased automation will be developed in the future.

## Data Availability

Not applicable.
